# Combination Impressions for Accurate Fitting Dentures*Reprinted from *Dental Digest* for December, 1918, and January, 1919.

**Published:** 1919-04

**Authors:** Victor H. Sears


### COMBINATION IMPRESSIONS FOR ACCURATE FITTING DENTURES *

BY VICTOR H. SEARS, D.D.S.

### FULL UPPERS

Select a clean, smooth, bright metal tray, a little larger than the mouth would indicate for an ordinary plaster impression. The tray should conform to the arch of the vault along the posterior margin and should extend well back over the tuberosities, and be at least one-eighth inch short of all muscular attachments on buccal and labial sides.

The correct length of a denture posteriorly will depend upon the location of the junction of the hard and soft palates as well as upon the flexibility of the anterior ridge. In all full cases the posterior margin should end on soft tissue; that is to say, the denture should end back of the hard palate. The more flexible the anterior alveolar ridge, the farther back will it be necessary to

* Reprinted from *Dental Digest* for December, 1918, and January, 1919.

extend the plate. In an extremely soft ridge case the plate should extend as much as one-fourth inch upon the soft palate and press firmly into this yielding tissue.

The junction of the hard and soft palates is located by either of two methods. First, by the foveola palatina, two little depressions just lateral to the median line at the junction of the hard and soft palates, or it may be located by having the patient open the mouth wide and saying "Ah" two or three times. It will be noticed that at a certain line the loose soft palate will be elevated every time a vocal effort is made. The line at which this soft, vibrating tissue raises and lowers corresponds, for our purpose, to the posterior border of the hard palate.

Shape a piece of high-fusing modeling compound to the tray. The black impression-tray compound suggested by Dr. Rupert Hall is well suited to this purpose. Proceed as in taking an ordinary compound impression, except that instead of holding the tray by the handle, place the index finger under the center of the tray and apply steady but light pressure upward or upward and slightly backward; this procedure will automatically secure equal pressure on both sides.

Chill the compound and remove the impression from the mouth. Trim away any excess compound on buccal and labial sides as well as along the posterior margin. Do not remove the compound from the tray yet.

Locate the hard areas. This can be done only by firm pressure upon every part of the vault. Generally the hard areas will be found to occupy a position corresponding to the old style airchamber, but there may be other hard places present. All hard areas including the rugae should be generously relieved in the compound tray at this stage. Too much relief at this stage is much better than not enough. Be sure to scrape the center enough so that the impression will not rock when biting pressure is applied. A sharp scraper is an excellent instrument for making relief in the compound.

Insert the impression into the mouth again and test for rocking. If, when firm pressure is applied in the bicuspid region, first on one side and then on the other, the impression does not rock, it may be assumed that the relief has been sufficient.

Separate the metal tray from the compound impression tray thus formed, being careful to keep it intact.

There should now be enough suction in the impression to hold it in position while taking the bite. Do not heat nor in any way change the palatine portion of the impression during the subsequent steps. This part is to serve as a key in seating the impression accurately to position.

Dry the occlusal portion of the compound tray thoroughly and add some sizzling hot compound to build up the occlusal plane. The compound for this purpose need not be high-fusing, but must become brittle at mouth temperature. The entire occlusal plane may be built up by adding the hot compound from the flame, but if much is to be added it is best to take a roll of plastic compound from the hot water pan. Very little is added in the molar region.

In building up the occlusal plane, both sides should be built up equally, and the compound should be equally soft on both sides so that the same amount of resistance will be offered to the muscles of mastication on both sides. If more resistance is offered to the muscles on one side than on the other, the muscles will not contract equally and a strained condition will result.

Dip the newly added hot material into warm water to prevent burning the patient's lower lip and quickly carry the impression to the mouth, seating it to position. During the process of adding and heating on the occlusal side, do not allow the heat to penetrate to the palatal side and distort the palatal fit.

As soon as the impression is properly seated, instruct the patient to quickly bring the lips together in the rest position and swallow. If the mandible does not move upward far enough, instruct the patient to swallow again and again until the correct relation has been established. The secret of success at this stage is not to let the patient know that your purpose is to register the bite. Say "Hold the impression up with the lower jaw," or "Keep the lower teeth against the impression so that it can not fall down," but under no circumstance suggest biting.

Have the patient hold the impression in place with the lower jaw until the compound has reached the brittle stage. The bite is then completed. Chill in cold water.

If the compound on the occlusal surface was distributed so that the resistance was equal on both sides, and if the compound was soft enough the normal relation was registered. However, the bite should be tested in order to be certain that it is correct. The test is made in the following manner:

With a sharp knife obliterate all of the bite marks thus taken except the very tips of the depressions made by the lower cusps.

Carry the impression to position in the mouth, and have the patient snap the lower teeth against the brittle compound two or three times in quick succession. If the bite is correct the patient can perform this act accurately each time, the impression will not shift as the teeth strike the occlusal plane, and the sound will be a solid one of all cusps hitting at one time. If, however, the patient has protruded the mandible in the first instance, or if a strained bite has been taken, the shifting of the tray can be both seen and felt when the lower cusps strike the occlusal plane. If strained, the patient will feel that one side strikes harder than the other. Also by listening carefully it will be heard that the cusps strike first on one side and then on the other, or there is a sound of sliding to position.

If it is found that the bite has been strained on the right side, for instance, and the right cusps strike the occlusal plane first, remove the impression from the mouth and with a sharp knife shave off a little from the right side of the occlusal plane. Now heat the whole occlusal surface to a depth of about one-eighth inch, carry the impression once more to position and have the patient bring the lips together and swallow. When the compound has been chilled, obliterate the depressions again, except the very tips of the cusps, and test as before. Having verified the bite the impression may then be completed.

The ideal way of constructing a denture is to take the impression with the buccal and labial rim the same height and thickness as the proposed denture, so that no changes will be necessary on the completed denture. To determine the exact height, proceed as follows: Cut the entire buccal and labial rim of the impression down nearly to the crest of the alveolar ridge. Build up first the right side of the rim with medium fusing compound. For the purpose of muscle-trimming, the compound should not be high-fusing but should remain brittle at mouth temperature.

Use the flame in building up the rim but be sure to immerse the compound in water of 150-160 degrees F. to prevent burning the patient.

When the right side of the rim has been built up in the manner described and the added compound is still soft, especially at the very edge, seat the impression to position. Carry it into the mouth, left side first, holding the lip and cheek high on the right side so that the soft compound will not be bent over in carrying it to position. Have the patient hold the impression up firmly in place with the lower teeth and muscle-trim the softened margin by alternately protruding the lips as in whistling and drawing them back as in smiling. This movement performed two or three times while the compound is in the flowing stage will cause the muscular attachments to cut into the soft mass and automatically establish the correct height of the rim. The excess compound thus turned down on the buccal and labial should be cut away, but do not alter in any way the margin of the rim. The facial contour may be restored in compound at this time.

Heat the other side in the same manner and carry it to place in the mouth, cool side first. Muscle-trim this side and trim off the excess. It may be necessary to heat the rim in the region of the six anteriors and have the patient muscle trim the front part of the impression at the place where the two halves of compound were added. After a little practice it will be found possible to add the entire rim and have the patient do all of the muscle trimming at one operation.

The final step in the making of the compound impression tray is known as "post-damming." Dry the palatine surface of the compound and place a thin roll of low-fusing wax across the posterior margin upon the palatine surface. The black wax upon which Trubyte Teeth are carded is ideal for this purpose. Seal this to the tray, and while the wax is still warm, carry the impression to position in the mouth. Have the patient bring the lower jaw into contact with the occlusal plane and press the impression firmly against the tissues so that the pressure caused by the wax will be equalized. The object of this wax is to seal the posterior margin against ingress of air. If the anterior ridge is firm, very little post-damming will be necessary.

Cutting from the inside, trim the buccal and labial rim all around to a sharp edge. This will give the necessary bulk to the plaster along the edge of the impression. Roughen the palatine surface by any means so that the plaster will stick to it.

Sift some model plaster into a clean dish and be certain that the plaster bowl is perfectly clean, so that no lumps or particles of plaster will be in the mix. A

small particle of any kind might prevent the impression from going accurately to position.

The plaster bowl is first moistened. Then fill a tablespoon with as much water as it will hold and pour this into the bowl. Stir a pinch of powdered potassium sulphate in the water to hasten the setting. Sift three level tablespoonfuls of model plaster into the solution and spatulate until the mix is smooth.

Dip the compound impression tray into water to prevent the plaster from hardening too quickly by contact with a dry surface. Place the soft plaster in the compound impression tray, making sure of an excess at the tuberosities and in front.

Quickly place the occlusal surface of the compound tray on the lower teeth so that the cusps rest into their corresponding depressions on the occlusal plane. Instruct the patient to bring the impression tray *fully* to position against the upper arch by closing the mouth. Hold the upper lip out labially while the patient is closing. As soon as the mandible has been brought up as far as it will go, notice whether the face has the proper contour. If there is excessive fullness at any place, light pressure on the cheek or lip will force the plaster out of the way. Have the patient hold the impression *firmly* in place until the plaster has set.

To remove the impression, instruct the patient to close the lips and fill the mouth with air. This should cause the impression to fall down without breaking. If this fails, inject a little water under the impression to break the adhesion.

Occasionally it may be necessary to make more than one attempt at securing a perfect impression. If so, all of the plaster should be removed from the compound impression tray or another very thin mix may be used over the first one. If the latter method is used, be sure to soak the impression in clean water before adding the second wash of plaster. In either event, the impression should be examined to discover if any place on the compound tray presses too hard against the tissue. This is indicated by the dark modeling compound showing through the white plaster. Any such dark spots should be scraped before a second attempt is made.

Wipe the roof of the mouth free from mucus. Moisten the point of a soft indelible pencil and outline the hard areas on the mucous membrane. The extent of the hard areas can be very accurately mapped out on the impression by outlining them in the mouth with an indelible pencil and then inserting the impression once more to position. The indelible pencil markings will thus be transferred to the plaster impression.

Relieve the hard areas with sandpaper or a sharp scraper. The rugae can best be relieved with a discoid.

The impression, as soon as dry, should be given a very thin coat of sandarach varnish. This will serve to prevent the thin areas of plaster from leaving the compound.

In taking the bite by this method it is, of course, necessary to take a separate impression of the lower teeth so that the cusps of the lower teeth may be made to fit into the depressions on the occlusal plane.

### SOFT RIDGE UPPER

A type of case which has given much trouble when the ordinary methods of impression taking are used, but which can be successfully handled by this technic is the one in which the anterior alveolar ridge is flexible. The technic varies somewhat from that employed for the firm ridge.

It is obvious that in a case of this type, the soft ridge will yield under biting pressure applied in the incisor region. It will be remembered that just posterior to this soft, yielding ridge there is a more solid area supported by a bony foundation. For this reason, every time the soft anterior ridge yields under such biting pressure, the unyielding area acts as a fulcrum upon which to force down the posterior part of the plate. It is therefore clear that if the denture is to remain in contact with the vault and thereby prevent the ingress of air when the posterior margin is being forced downward, this part must be firmly imbedded in the soft, yielding tissue posterior to the hard palate. In general, the greater the tipping movement is, the greater must be the pressure upon the soft palate. Also, the softer the anterior ridge, the farther back must the denture extend. It should be remembered that extending the plate upon the soft palate will often cause nausea, unless the extension covering this sensitive part presses firmly upon the tissue.

In taking the first modeling compound impression the ridge will be forced labially, but it is obvious that for the patient's comfort, as well as for the greate stability of the denture, the impression should be completed with the soft ridge in its normal position.

Just lingually to the anterior ridge cut the compound away generously, before removing the compound from the metal tray. It will be seen that in taking the first impression, there must be enough compound in the anterior part of the vault to allow for this trimming without cutting through the impression. To prevent the soft plaster from forcing the flexible ridge labially, a little soft wax may be filled into the front part of the impression before the plaster wash is used.

Complete the impression as though it were an ordinary full upper, except that the posterior margin will be extended farther posteriorily, and more post-damming will be necessary.

### FULL LOWERS

The technic for lowers is substantially the same as that used for full uppers, except that that part of the impression which covers the masseter muscle should be completed with that muscle in contraction, as explained in the two following paragraphs. The compound impression tray should extend posteriorly upon the ridge as far as the ramus, but the high-fusing compound should not extend very far bucally, labially, or lingually. Do all of the muscle-trimming in medium-fusing compound. The labial rim should be trimmed until the lower lip does not raise the impression when the mouth is slightly opened.

Heat the buccal rims of compound on both sides in the region of the masseters. The compound should be soft as far as the crest of the ridge, and as far anteriorly as the bicuspid region. When the compound is soft, seat the impression upon the ridge and have the patient bite upon it until the compound has set. The impression now fits and bears evenly upon the tissues under working conditions. If much excess is turned up by the muscular contraction, this excess should be trimmed away.

After correcting the impression in the region of the masseters the next step is to secure "suction." As is the case with uppers, "suction" on the full lowers depends upon fitting the margins of the denture against yielding tissue, thus preventing the ingress of air.

The method advocated by Dr. Hall requires that the lingual rim of the denture should fit over the mylo-hyoid ridge, imbedding itself into the floor of the mouth. Dr. Hall believes that the undercut frequently present below the mylo-hyoid ridge is a valuable aid in holding down the lower denture. The fact that patients are wearing lower dentures made in accordance with this principle is proof that some mouths will tolerate this long lingual rim. However, the muscles attached to the mylo-hyoid ridge must be stretched over the lingual rim of the denture if this rim is extended lower than the muscular attachments, and the experience of operators in general tends to show that some mouths will not tolerate this condition.

Mr. Supplee teaches muscle-trimming of the lingual margin of lower impressions. If the lingual margin be very soft, and the patient be allowed to hold the impression firmly against the upper and swallow, it will be noticed that there is a definite angle formed by the floor of the mouth and the lingual aspect of the alveolar ridge. The lingual margin of the impression is trimmed away so that the plate will not extend below this angle. Frequently it will be found necessary to trim more than is indicated by this procedure.

The method developed by Dr. Tench is one generally suited to all kinds of full lower cases. It consists in ending the lingual margin of the denture just at the crest of the mylo-hyoid ridge or very little below it. This assures clearing the attachments of the mylo-hyoid muscles. Dr. Tench builds enough low-fusing wax along the lingual margin to seal it against leak. Sometimes this addition is necessary only back of the six lower anteriors. Sometimes it is necessary to dam the posterior margin of the denture at the crest of the ridge before good suction can be developed.

If the impression is smooth on the ridge side and has good "suction," a cast may be run in it without further preparation. If, however, there are any large creases or other defects visible, the impression should be completed with a plaster wash and under biting pressure.

Whenever the ridge is soft or thin and sharp, it will be necessary for the comfort of the patient to cut the trough in the compound impression tray deep enough to prevent the compound from coming in contact with the crest of the ridge. The compound impression tray will thus rest on both sides of the ridge. The thin plaster wash will fill in the trough and complete the impression without distorting the ridge.

